# Take home messages from the implementation of the primary health care measurement and improvement (PHCMI) initiative in the WHO Eastern Mediterranean Region (EMR)

**DOI:** 10.1017/S1463423625000179

**Published:** 2025-03-14

**Authors:** Karen Kinder, Hassan Salah, Hagar Azab, Hamid Ravaghi, Henry Doctor, Roberta Tosques, Arash Rashidian, Awad Mataria

**Affiliations:** WHO Regional Office for the Eastern Mediterranean, Cairo, Egypt

**Keywords:** Eastern Mediterranean Region (EMR), performance assessment, PHC measurement and improvement (PHCMI), PHC performance initiative (PHCPI), primary health care approach (PHC), universal health coverage (UHC)

## Abstract

**Aim:**

To summarize the findings from the PHC Measurement and Improvement (PHCMI) initiative in the World Health Organization WHO Eastern Mediterranean Region (EMR) and highlight key lessons to build PHC-oriented health systems.

**Background:**

To support countries in enhancing their commitment to the primary health care (PHC) approach, the WHO Regional Office for the Eastern Mediterranean (EMRO) established the PHCMI initiative in 2019 to evaluate PHC performance and identify priorities for improvement.

**Methods:**

Building on the experience of the global PHC Performance Initiative (PHCPI), PHCMI updated the master indicator list through iterative processes and reflecting country priorities. Progress in five domains: system/structure, inputs, processes, outcomes, and impact was assessed. Existing policy documents, data sources, and key informant interviews were used in the assessment.

**Findings:**

Of the 21 countries and one territory, 14 participated in the assessment including Afghanistan, Bahrain, Egypt, I.R. of Iran, Iraq, Jordan, Lebanon, Libya, Morocco, Oman, Pakistan, occupied Palestinian territory, Qatar, and Yemen. Despite the observed heterogeneity in PHC implementation, most countries reported that public primary care services were acceptable, accessible, and affordable. Strong leadership, the existence of PHC-related national health policies, and the engagement of stakeholders were key success factors.

Areas for additional attention include the need to increase investment in PHC, increase the PHC workforce, enhance equitable distribution of the workforce, expand training of primary care clinicians, improve health information systems, and inform the population on the benefits of preventive care. Health systems should be more tailored to the PHC approach and encouraged towards a more holistic view of the PHC approach.

The PHCMI initiative provides a foundation for an open discourse for advancing universal health coverage (UHC) and health security in EMR and re-orienting health systems towards the PHC approach.

## Background

The Eastern Mediterranean Region (EMR) includes 21 countries and one territory and accounts for almost 9% of the world’s population (WHO Regional Office for the Eastern Mediterranean, 2020). The heterogeneity of the region is underscored by the variance in income level: there are six high-income, three upper-middle-income, eight lower-middle-income, and five low-income countries or territories (World Bank, 2022). In addition, multiple conflicts and humanitarian crises as well as natural disasters have resulted in significant internally displaced, refugee, and migration populations, which impact the ability to deliver essential health services and achieve universal health coverage (UHC) (WHO Regional Office for the Eastern Mediterranean, EM/RC69/R.2, 2022).

In 2021, the regional average for UHC Service Coverage Index (SCI) stood at 57 (out of 100), compared to the global average of 68 (World Health Organization and World Bank, 2023). There are also significant disparities between countries according to their income and political stability. It is widely accepted that a strong health system foundation based on the primary health care (PHC) approach is essential to achieving UHC as well as ensuring health security for all (WHO Regional Office for the Eastern Mediterranean, EM/RC69/R.2, 2022), (WHO Director General EB152/5, 2023) (World Health Organisation, 2023). Notably, it has been estimated that 90% of the recommended interventions to achieve UHC can be delivered at the primary care level contributing to 75% of potential health gains under the Sustainable Development Goals. (WHO Director General EB152/5, 2023).

**Figure 1.**
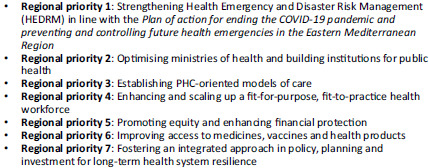
Seven regional priorities for building resilient health systems towards UHC and health security.

A recent World Health Organization (WHO) Eastern Mediterranean Regional Committee resolution (WHO Regional Office for the Eastern Mediterranean, EM/RC69/R.2, 2022) put forward seven priorities for building resilient health systems to achieve UHC and ensure health security in EMR as shown in Figure 1. One of these priorities singles out the need to establish PHC-oriented models of care. Other priorities address the need to integrate essential public health functions with primary care services as well as other multisectoral aspects of the PHC approach. The resolution stressed the need to pivot towards a health system oriented towards PHC in EMR (WHO Regional Office for the Eastern Mediterranean, EM/RC69/R.2, 2022).

Delivery of primary care services in the Region is largely characterized by disparity in health service delivery, where 70% of outpatient services are provided by the private health sector (WHO Regional Office for the Eastern Mediterranean, 2014), 93% of primary care facilities are managed by generalist (WHO Regional Office for the Eastern Mediterranean, 2016), and 1 out of 10 patients admitted to public hospitals experience adverse events (Letaief *et al.*, 2021).

In addition to existing challenges, the COVID-19 pandemic exposed gaps in the linkages between primary care services and essential public health functions, which are critical to ensuring health security. Despite its impact on the ability to meet the health needs of populations, the recent pandemic served to reinforce the value of multisectoral action and community empowerment, both of which are aligned with the PHC approach.

When affirming the Declaration of Astana (World Health Organization and the United Nations Children’s Fund, 2019), EMR Member States recognized that components of PHC needed to be updated to respond to ongoing and new health and health system challenges, as well as to take advantage of new resources and opportunities for success in the 21st century. The recent pandemic underscored the need to invest in a PHC approach to strengthen health system’s resilience to ensure health security. This recognition has inspired global commitment towards the reorientation of health systems towards the PHC approach.

In recent years, the WHO Regional Office for the Eastern Mediterranean (EMRO) has launched several initiatives driven by this acknowledgement, including: the PHC Measurement and Improvement (PHCMI) initiative, a regional UHC priority benefit package (UHC-PBP) (Mirza *et al.*, 2018) (Salah *et al.*, 2021) (Mataria *et al.*, 2023), a regional professional diploma in family medicine, online training for PHC physicians on COVID-19 (WHO EMRO, Role of PHC in COVID, 2020), enhanced effective engagement with the private health sector (Iqbal *et al.*, 2024) (Muhjazi *et al.*, 2024) (WHO Regional Office for the Eastern Mediterranean, private sector engagement), and the development of PHC-oriented models of care (WHO Regional Office for the Eastern Mediterranean, PHC MoC).

### The primary health care approach

WHO describes PHC as ‘a whole-of-society approach to health that aims equitably to maximize the level and distribution of health and well-being by focusing on people’s needs and preferences (both as individuals and communities) as early as possible along the continuum from health promotion and disease prevention to treatment, rehabilitation and palliative care, and as close as feasible to people’s everyday environment’ (World Health Organization and the United Nations Children’s Fund, 2018).

The PHC approach is defined by three components: 1) integrated health services to include primary care services and essential public health functions; 2) addressing the broader determinants of health through multisectoral policy and actions and 3) empowering individuals, families, and communities to take charge of their own health (World Health Organization and the United Nations Children’s Fund, 2018) (Iqbal *et al.*, 2024). Overall, primary care in the Region has been disease-oriented rather than people-centred (WHO Regional Office for the Eastern Mediterranean, EM/RC69/R.2, 2022). Given PHC’s component of multisectorality, there is a need to pivot away from vertical programmes focused on diseases to a holistic view of people and populations, which implies that other sectors of society need to be involved in the solution.

The 2018 Global Conference on Primary Health Care renewed its commitment to PHC, adopting a modern perspective that resulted in the ratification of the Astana Declaration, which clarified and emphasized the importance of the ‘PHC Approach’ (World Health Organization and the United Nations Children’s Fund, 2019). Supporting documents included ‘A Vision for primary health care in the 21^st^ century’ (World Health Organization and the United Nations Children’s Fund, 2018), which made the investment case for PHC, while the ‘WHO PHC Operational Framework’ provided guidance to take a country from vision to action (World Health Organization and the United Nations Children’s Fund, 2020). A series of technical documents and country case studies were made available to assist Member States as they embarked on reforming their health systems.

### The primary health care measurement and improvement (PHCMI) initiative

**Figure 2.**
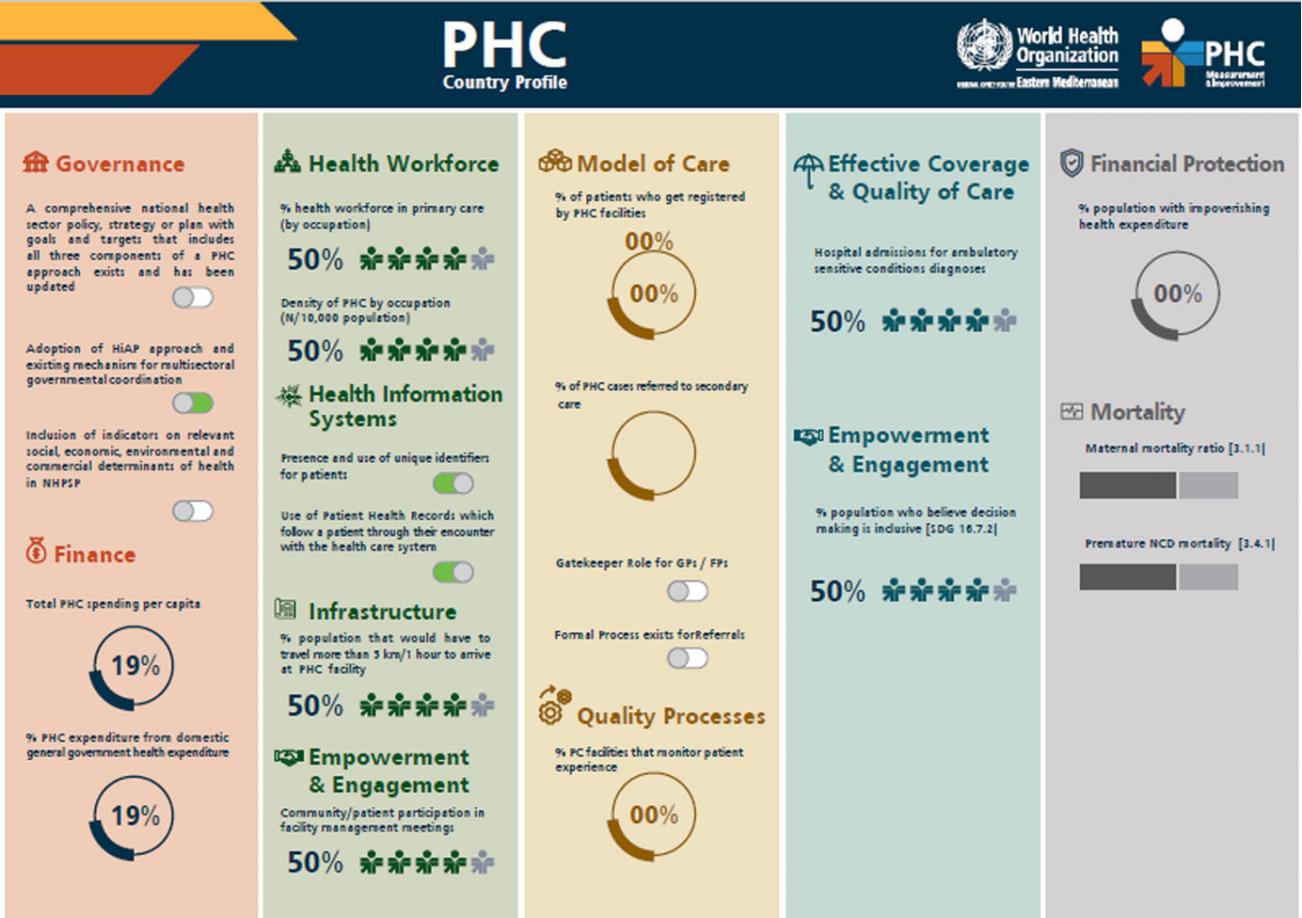
Generic PHCCP dashboard.

**Figure 3.**
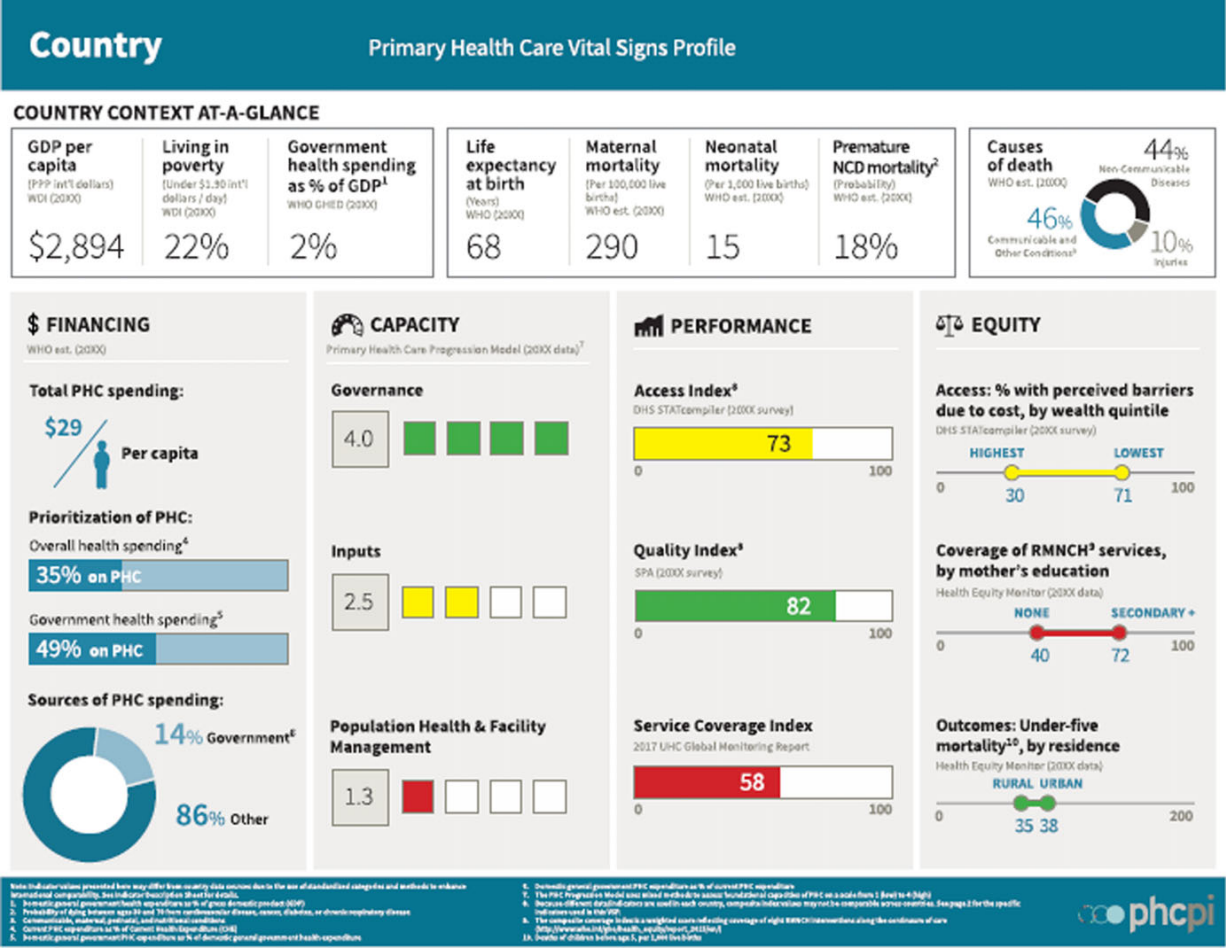
Generic VSP dashboard.

To support Member States in fulfilling their commitments made in the Astana Declaration, WHO EMRO established the PHCMI initiative in 2019, in collaboration with the United Nations Children’s Fund (UNICEF), the World Organization of Family Doctors (WONCA), and the PHC Performance Initiative (PHCPI), with support from the Bill and Melinda Gates Foundation, to evaluate PHC performance in the region; determine focus areas to improve the region’s delivery of primary care services and essential public health functions; and reorient the region towards the three components of the PHC approach (Muhjazi *et al.*, 2024) (WHO Regional Office for the Eastern Mediterranean, PHCMI, n.d.). The objectives of PHCMI were to develop a common approach to assess PHC performance; to aggregate key PHC data from the region and at country level; to create tools such as the PHC Country Profiles while leaning on the PHCPI’s (PHCPI, home page, n.d.) ‘Vital Signs Profiles’ (PHCPI, VSPs, n.d.); to highlight progress and challenges to improving PHC performance; and to integrate PHC improvement plans into routine policy and service delivery processes (WHO Regional Office for the Eastern Mediterranean, PHCMI, n.d.). Generic versions of the PHC Country Profiles and the Vital Sign Profiles can be found in Figures 2 and 3.

The aim of this paper is to highlight the findings and lessons gained from the PHCMI initiative and bring forward potential steps for moving forward to build PHC-oriented health systems in the EMR.

## Methods

As part of the PHCMI initiative, a framework was created containing five domains: system/structure, inputs, processes, outcomes, and impact as well as a master indicator list (Annex 1), which identifies indicators to measure achievement in those domains. It built on the PHCPI Framework (PHCPI, home page, n.d.), the PHCPI Progression Model (PHCPI, Progression Model, n.d.), the PHC Operational Framework (World Health Organization and the United Nations Children’s Fund, 2020), existing regional efforts to strengthen PHC, and established WHO PHC quality indicators (WHO Regional Office for the Eastern Mediterranean, quality and patient safety, n.d.).

Assessment relied on locally available data, knowledge, and evidence, including information obtained through document review, quantitative data capture, and qualitative interviews with key informants with local PHC expertise. Routinely collected data including facility surveys; routine assessments of health information systems, individual patient records, and electronic medical record systems; patient and health care provider surveys; population-based and household surveys; as well as civil registration and vital statistics systems were analyzed to determine what quantitative indicators could be measured. Global databases were also accessed to augment the available data. These sources were selected following several rounds of review by global PHC monitoring and evaluation experts. When possible, globally comparable data sources were prioritized to enhance international comparability, fostering accountability, and cross-country learning. For more details on the indicators, metadata, or measurement challenges, readers can refer to the Global Health Observatory at https://www.who.int/data/gho/data/indicators.

The PHC Progression Model (PHCPI, Progression Model, n.d.) is a mixed method tool used to capture information through qualitative assessments involving interviews with multiple stakeholders. The Progression Model enables standardized, systematic assessment of basic areas of PHC performance and core PHC capacities, including governance and leadership, adjustment to population health needs, service delivery inputs, population health management capacity, and the organization and management of health facilities (PHCPI, Progression Model, n.d.).

To facilitate the capture of data and better structure the findings, a PHC Country Profile dashboard was created to accompany the PHCPI Vital Signs Profile.

The initiative’s assessment of the performance of PHC consisted of three phases: planning, measurement, and improvement.

During **the planning phase**, following a formal launch of the PHCMI initiative on World Health Day 2019, the initiative partners conducted a thorough stakeholder analysis as the first step in implementing PHCMI upon approval from the relevant Ministries of Health to commence the initiative. The stakeholder analysis systematically gathered and analyzed qualitative information to determine whose interests should be considered when implementing a policy or programme; defined the desired outcomes and objectives of the assessment, its scope, and parameters; established effective communication and collaboration between all stakeholders; and developed a workplan that facilitated the regular monitoring of progress taking into consideration each country’s context.

To commence the **measurement phase**, in July 2019, WHO EMRO conducted the first regional consultative meeting on PHC for UHC (WHO Regional Office for the Eastern Mediterranean, 2019). With twenty countries in attendance, the three pilot countries (Egypt, Pakistan, and Jordan) presented their preliminary findings. An initial master indicator list comprising comprehensive indicators of PHC performance was developed by WHO in consultation with PHCPI, as well as feedback from pilot country experiences, leaning on the evolving indicators list being developed for the PHC Measurement Framework and Indicators and under consideration of the EMR setting (World Health Organization and the United Nations Children’s Fund, 2022).

Following the selection of indicators by WHO EMRO, several missions and online training sessions were conducted to discuss and review the master indicator list and propose alternative indicators based on a country’s specific context given it was not always possible for a country to collect data for some selected indicators and, at some cases, indicators were not considered relevant. The list of alternative indicators was reviewed and finalized in collaboration with PHCMI country focal points. After finalizing the master indicator list, the PHCMI country focal point liaised with other team members and stakeholders to identify what data were available or needed to be collected during the measurement phase. After identifying which information was available, the PHCMI country focal point contacted key informants to collect data.

Some quantitative data was already available while the collection of other indicators required interviews with key informants. A part of the PHCMI assessment was informed by the PHCPI Progression Model qualitative assessment tool, the results of which provided a basis for countries to track their progress in improving capacities for stronger performance. Each aspect of PHC capacities assessed through the Progression Model required the collection of relevant data, which included key informants’ interviews. While different kinds of relevant data were used, some were mandatory for the assessment to be considered valid. Country focal points were advised to interview MoH staff who were directly related to PHC or data collection. For aspects related to governance and leadership, interviews with non-governmental key informants were mandatory. Non-governmental informants were working in relevant civil society organizations, NGOs, and academia. Ten countries – Afghanistan, Bahrain, Egypt, Jordan, the Islamic Republic of Iran, Iraq, Morocco, Oman, Pakistan, and Qatar – finalized the Progression Model. There were differences in the implementation process as countries employed multiple approaches and strategies to finalize the project.

The Progression Model was implemented, in full collaboration with PHCPI experts, through a joint internal/external assessment, consisting of an internal self-assessment conducted by an in-country team and an external validation completed by the Regional Office Progression Model focal point. The goal of external validation was to ensure results were evidence-based and calibrated across countries.

Once all data were collected, validated, and approved by the Ministry of Health, a formal clearance process was initiated. If any discrepancies were identified by WHO at the country, regional, or global level, they were addressed before data was made public. Finalized data was used to inform the PHC Country Profiles and the Vital Signs Profiles for publication.

Using the lessons learned from the measurement phase, the initiative moved into the **improvement phase**. In December 2022, the 2^nd^ PHC for UHC meeting with 140 participants from 19 countries and PHC regional partners was conducted. In addition to providing country experiences, countries identified priority areas for improvement. For some countries, this involved incorporating PHCMI findings into existing reforms, using PHC Country Profiles as evidence for driving policy dialogue, and/or engaging country leadership and partners in collaborations that strengthened PHC.

## Findings

In its first regional analysis of PHC, the Regional Committee for the Eastern Mediterranean, held in October 2022, acknowledged the gaps in health care systems exposed by the recent pandemic affecting the ability to achieve UHC and health security (WHO Regional Office for the Eastern Mediterranean, EM/RC69/R.2, 2022).

Even before COVID-19, the Region recognized the need to secure high-level political commitment; strengthen the capacities of ministries of health and health-related institutions; adopt workable models of care; reduce the share of out-of-pocket payments and enhance financial protection; develop a balanced, skilled, and motivated health workforce; reinforce health information systems; and improve access to medicines and essential technologies and strengthen medical supply chains (WHO Regional Office for the Eastern Mediterranean, EM/RC59/R.3, 2012).

Out of the 21 countries and one territory in the region, 20 participated in the initiative and 14 provided the data included in the Regional Assessment upon endorsement by the respective Ministries of Health (Afghanistan, Bahrain, Egypt, I.R. of Iran, Iraq, Jordan, Lebanon, Libya, Morocco, Oman, Pakistan, occupied Palestinian territory, Qatar, and Yemen) (WHO Regional Office for the Eastern Mediterranean, country profiles, 2017–2020). The lack of timely endorsement by the Ministries of Health prevented the inclusion of the remaining six countries’ profiles in the manuscript.

Despite all the efforts, most countries had data gaps and missing information for indicators, and none of the countries provided data for all the indicators in the master indicator list. (Annex 1). All PHC Country Profiles and Vital Sign Profiles can be accessed here: https://www.emro.who.int/uhc-health-systems/access-health-services/country-profiles.html

Regarding the overall challenges facing the PHC approach, 29% of countries noted poor engagement with the private health sector, 17% noted health workforce issues, programme fragmentation, and problems facing quality measurement of services, and 5% noted a need for more consistent essential health services.

In the Region, based on the figures reported in the PHC Country Profiles in 2021, total annual spending per capita on PHC ranged from $28 USD (Afghanistan) to $310 USD (Qatar), with a median of $183. The median percentage of PHC expenditure from general government health spending was 27%, with actual figures varying from 1% in Afghanistan to 49% in Egypt.

Committed leadership and the need for strong continued political support stood out as major strengths in relation to PHC performance improvement. There was an acknowledgement of PHC in national health strategies, quality standards, and reform in all 14 responding countries. Social, economic, environmental, and commercial determinants of health were included in the national health policies of nine of the 10 countries that submitted replies. All but one of the 10 responding countries indicated they had adopted a Health-in-All-Policies approach and had a mechanism for multisectoral governmental coordination. Other areas of strength reported included the engagement of stakeholders, including the community, in policy and strategy development.

A majority of countries reported that public primary care services were acceptable, accessible, and affordable. Immunization, water sanitation, and infection control in facilities were reported as being widely implemented in most countries. However, several countries mentioned limited availability of essential medicines.

While some countries reported that primary care workers were qualified, others suffered from a shortage of competent primary care staff and effective family medicine services due to the limited number of family physician graduates. The density of the health workforce providing comprehensive primary care services differs by country and occupation as can be seen in Table 1. The number of consultations per health worker per day by country can be seen in Table 2 and selected input, processes, and impact indicators can be found in Table 3.

**Table 1.** Density of primary care health workforce by occupation per 10 000 population

	Physicians	Nurses	Dentists	Pharmacists	Midwives	Allied health professionals
Afghanistan	na	na	na	na	na	na
Bahrain	2.5	4.1	0.6	na	na	na
Egypt	2.56	6.17	1.13	2.18	na	na
I.R. of Iran	1.3	na	na	na	3.2	15.2^*^
Iraq	1.49	6.3	1.43	0.86	na	8.3^†^
Jordan	1.12	2.38	0.53	0.23	0.76	na
Lebanon	3.62	1.13	8.07	na	1.65	na
Libya	7	38.7	7.7	4	0.8	na
Morocco	1	1.6	na	na	0.6	na
Oman	4.83	11.41	0.54	1.92^‡^	na	na
Pakistan	na	na	na	na	na	na
oPt	na	na	na	na	na	na
Qatar	3.19	7.29	0.78	1.39	na	na
Yemen	na	na	na	na	na	na

Data collected 2017–2020 from PHCCPs.

* Includes community health workers.

† Includes paramedical staff.

‡ Includes pharmacy assistants.

na = not available.

**Table 2.** Number of consultations per health worker per day

	Physicians	Nurses	Dentists	Pharmacists	Midwives	Allied health professionals
Afghanistan	na	na	na	na	na	na
Bahrain	30.5	64	9	na	na	11^*^
Egypt	na	na	na	na	na	na
I.R. of Iran	35	na	5	na	58	27^†^
Iraq	16.5	0.6	1.6	na	na	na
Jordan	24.2	7.8	na	na	na	na
Lebanon	27.8	na	na	na	na	na
Libya	na	na	na	na	na	na
Morocco	29	19	na	na	na	na
Oman	na	na	na	na	na	na
Pakistan	na	na	na	na	na	na
oPt	na	na	na	na	na	na
Qatar	16.3	17.9	na	na	na	na
Yemen	na	na	na	na	na	na

Data collected 2017–2020 from PHCCPs.

na = not available.

* Physiotherapists.

† Healthcare expert.

**Table 3.** Selected input, processes, and impact indicators

	% popln registered at PC facility	% cases referred to secondary care	Annual utilization rate per capita	Vacancy rate in PHC (in %)	% doctors specializing in family medicine	% popln with normal BP^*^
Afghanistan	na	1.25	2.5	53.8 doctors; 22.5 nurses	na	39
Bahrain	54.3	6.27	4.9	13.4	72	66
Egypt	na	na	0.5^†^	11.7	na	75
I.R. of Iran	95	8.4	4.1	19% doctors; 33% behvarz	83	60
Iraq	na	na	1.6	57.8	13.7	50
Jordan	na	na	30	na	4.1	79
Lebanon	27	na	na	13	1.4	79
Libya	na	na	3	na	0.05	53
Morocco	35	8,4	0.6	4	3.5	71
Oman	na	na	3.4	0	10	66
Pakistan	na	na	1.2	na	na	38
oPt	na	na	2.1	na	1.1	64
Qatar	63	6,85	1.25	10.1	70	na
Yemen	40	na	na	na	na	na

Data collected 2017–2020 from PHCCPs and VSPs.

na = not available.

* Percentage of adult population with normal blood pressure is based on age-standardized estimates. These distributions are rescaled to provide finer resolution before inclusion in the index. Rescaled indicator = (X–50)/(100–50) × 100, where X is the prevalence of normal blood pressure. For more details see Tracking UHC: 2017 Global Monitoring Report.

† This value only refers to curative services in outpatient departments.

The density of primary care providers is important to assess access. Another measure of access is the percentage of the population that must travel more than 5 kilometers or 1 h to arrive at a primary care facility. It ranged from 56.6% in Afghanistan to none in Bahrain, though it should be noted only five countries were able to supply a value for this indicator.

A lack or limited use of any centralized health information system (HIS) across levels of care at both the national and subnational levels was also reported, especially in low-income countries. Gulf Cooperation Council (GCC) countries, however, reported strongly connected digital systems. Some countries reported the presence of multiple parallel HISs. Regarding major barriers in collecting primary care information, 27% of participating countries noted data availability, 24% mentioned unclear data routes, and 20% highlighted issues with overall data reliability and fragmentation. All nine countries that reported on the use of unique identifiers for patients had them, and all five responding countries reported using patient health records to follow patients through their encounters.

The model of primary care service provision indicators reflects the degree to which a country embraces the PHC approach. Among the five countries that reported the percentage of patients registered with a primary care facility, the range varied from 27% (Lebanon) to 95% (I.R. of Iran). Lack of shared patients’ records and a formalized referral system mechanism proved to be a challenge in many countries. Of eight countries reporting a formal process for referrals, seven cited general practitioners as having a gatekeeper role. In most countries, the provision of mental health services, as part of a primary care service package, was found to be limited and the quality of the services was inadequately assessed.

Community engagement is a core component of the PHC approach. All nine countries reporting indicated there was community and/or patient participation in facility management meetings. To assess patient-centred aspects, countries were asked what percentage of primary care facilities monitored patient experiences. Six countries responded, and the range varied from 15% (Morocco) to 100% (Qatar). The average was 64.5%.

As with the variance in inputs, there were differences in outcome measures. Life expectancy at birth ranges from 65.3 years (Afghanistan) to 80.7 years (Qatar), suggesting a correlation between PHC spending per capita and years of life. Premature NCD mortality varied from 11.3% (Bahrain) to 30.6% (Yemen). Maternal mortality ratio varied from a low of nine deaths per 100 000 live births (Oman) to a high of 638 deaths per 100 000 live births (Afghanistan). It was found that coverage of reproductive, maternal, newborn, and child health services ranged from 38% in Afghanistan – for mothers with no formal education – to 100% in Bahrain for all maternal educational levels. The average availability of the five tracer RMNCH services differed from 60% (Libya) to 100% (Bahrain, Lebanon, and Oman). The range in under-five mortality rates per 1000 live births between rural and urban settings remains an area of concern. Rural setting rates varied from 0 (Bahrain) to 83 (Pakistan), while urban settings ranged between 0 (Bahrain) to 56 (Pakistan) in the eight countries reporting.

*‘It was first ever initiative in the country to comprehensively assess primary health care, and from 360 degrees, and included assessing the system/structure, inputs, process, outcome, multisectoral approach, community empowerment and impact by using a single initiative (master indicator list) to set a baseline for the implementation of the Astana Declaration.’*

Afghanistan questionnaire response

### Use of PHCMI results at the country level

The implementation of the assessment resulted in a comprehensive picture of the capacities that must be present for a health system to work for all people. Information was not available on whether the findings were used in improving the quality of the services; however, a few countries used the findings to develop strategies for improvement.

The **Islamic Republic of Iran** used the PHCMI framework, from which the PHC Country Profiles were developed, in their development of a sub-national PHC measurement framework and country-specific indicators based on age groups as well as in the preparation of a dashboard for monitoring the indicators over a 10-year period (Rezapour *et al.*, 2023).

Weaknesses identified in **Pakistan’s** Vital Sign Profile assessment guided the prioritization of several actions such as: generating more evidence; implementing an essential package of health services; enhancing stakeholder coordination; strengthening governance structures, and introducing incentives to promote engagement of the private sector; prioritizing the capacity of the health workforce; enhancing further investment in the health sector; supporting the implementation of the health information system; and utilizing in-country public health and research institutions (World Health Organisation and the Pakistan Health Planning, System Strengthening and Information Analysis Unit in the Ministry of National Health Services, Regulations, & Coordination, 2022).

While **Morocco**’s data revealed strong national PHC leadership and accountability, data collected from sub-national directorates revealed a different picture. Staffing and budget shortages negatively impacted the sub-national ability to effectively manage the delivery of primary care services. The assessment led the government to take action to improve PHC leadership at all levels, with a focus on involving sub-national directorates in priority-setting exercises and strategic planning.

**Oman** realized that while it has a strong focus on monitoring health indicators, there is limited focus on factors that impact the indicators, making it difficult to identify what in the system results in specific outcomes. As a result of this realization, Oman plans to place more focus on processes within the system rather than the input and outcome indicators only in order to create improvement strategies. **Morocco** had a similar realization.

In response to the assessments, **Qatar** is establishing new services, including delivering medications to chronically ill and other high-risk patients via the postal service to improve access.

During **Iraq**’s assessment process, improvement measures were undertaken to support more comprehensive and effective data collection. During the assessment of the service delivery inputs, a list of essential consumable commodities recommended for primary care centres was compiled, allowing for the identification of gaps in essential supplies. A general recommendation to redirect more funds towards PHC-related goals was also followed by several specific recommendations based on identified areas for improvement.

## Discussion

The implementation and results of the PHCMI initiative underscored the heterogeneity of the region. Some similarities and trends did, however, emerge in relation to the impact of the initiative. Most countries reported that bringing together different data sources created an opportunity to holistically understand capacities in a way that challenged expectations, even for stakeholders embedded in the system. In some cases, gaps came to light for the first time. The process of conducting interviews with informants with diverse expertise resulted in a more accurate and wider picture of the situation in the country.

Other areas of strength identified included the existence of national health policies and strategies that reflect the three components of PHC and the engagement of stakeholders, including the community, in policy and strategy development.

Using insights gained from the measurement phase, countries were encouraged to identify priority areas for improvement and collaboration to address gaps, challenges, and weaknesses. The experience of countries during the PHCMI initiative highlighted the importance of supporting existing efforts to prioritize capacity building of MoH officials in primary care and leveraging the collection of data to improve reform efforts while identifying data gaps.

Additional opportunities identified in the governance domain included incorporating PHCMI findings into existing reforms and using the PHC Country Profiles as evidence to drive policy dialogue as well as engage country leaders and partners in collaboration aimed at PHC strengthening. The creation of a stronger system for priority setting at the national level, which allows communities to have a voice in resource allocation was also recommended.

Challenges also arose in relation to the limited political support in some countries and levels of expertise of key informants. In some cases, involved stakeholders did not have basic knowledge about public health and it was difficult for them to understand the topics presented in the assessment or to speak about the performance of the system. The training workshops helped in building their technical capacity. In other cases, responsible informants and stakeholders were reluctant to share information and evidence of low-performing systems.

In addition to the monitoring and evaluation of PHC, the integration of the PHC approach can be seen in other regional initiatives, including those directed at private sector engagement, more sustainable political support for strengthening the capacity of family medicine within regional institutions, recognizing the role of health workers in maintaining needed health services, establishing a benefit package for promotive, preventive, curative, rehabilitative and palliative services, and advocating for sustainable resources to ensure continued improvement and support (WHO Regional Office for the Eastern Mediterranean, UHC PBP, n.d.; WHO Regional Office for the Eastern Mediterranean, private sector engagement, n.d.; Mataria *et al.*, 2023).

In a Regional Committee Meeting, Members States endorsed a PHC Model of Care as the third regional priority for building resilient health systems to advance UHC and ensure health security in EMR (WHO Regional Office for the Eastern Mediterranean, EM/RC69/R.2, 2022). In addition, a regional initiative was developed with the goal of supporting Member States in developing PHC-oriented models of care reflecting one of the seven priorities outlined in a recent regional resolution (WHO Regional Office for the Eastern Mediterranean, EM/RC69/R.2, 2022). In line with the PHCMI Framework, PHC-oriented models of care rely on the PHC approach as outlined in the PHC Operational Framework (World Health Organization and the United Nations Children’s Fund, 2020) and is defined as the ‘conceptualization and operationalization of how services are delivered, including the processes of care, organization of providers and management of services, supported by the identification of roles and responsibilities of different platforms and providers along the pathways of care’ (World Health Organization and the United Nations Children’s Fund, 2020) (WHO Regional Office for the Eastern Mediterranean, PHC MoC, n.d.). In addition, EMRO drafted guidance for developing the minimum requirements of primary care facilities based on the national model of care (WHO Regional Office for the Eastern Mediterranean, Expert Consultation, 2023).

Although much work is being done in relation to PHC at the national and regional levels, it is important to acknowledge where efforts have not been focused and where a new approach may need to be considered. In the area of physical infrastructure, digital technologies for health, and monitoring and evaluation, there is little or no work currently taking place at the regional level. It is in these areas where a systemic approach to a PHC-oriented health system while ensuring it is a component of all health system strengthening efforts can facilitate achieving UHC.

The main recommendations on inputs addressed the density and qualifications of the workforce and included the need for training and capacity building of physicians, particularly the need to increase the number of family doctors, and nurses. Some countries disclosed a lack of proper implementation and/or the utilization of accredited points for continuous medical education for health care workers, an important factor in ensuring the sustainability of primary care service provision.

Weaknesses in most countries also included missing data on infrastructure, implementation of quality indicators and processes as well as outdated clinical guidelines which, even when available, were not always followed. It also emerged that perceptions of primary care and the experiences of service users were not always clearly evaluated. The need to focus on the ways services can be provided to reduce the burden of referrals elicited discussions of empowering individuals to self-manage their care, enhanced use of telemedicine and self-assessment software applications, and the enabling of virtual medical consultations with telephone follow-ups. Other priority areas uncovered included improving the measurement and collection of data on NCDs as well as ensuring the incorporation of NCD services into primary care services delivery. This is especially relevant given that NCDs are a leading cause of death in much of the EMR (Hammerich *et al.*, 2022),

While the creation of improvement strategies has begun, challenges remain including limited political commitment, health workforce supply issues, and scarcity of financial resources. Coordination of donors and other stakeholders is also suboptimal (WHO Regional Office for the Eastern Mediterranean, EM/RC69/R.2, 2022).

It was recognized that multisectoral coordination and collaboration are key to better responding to people’s needs, especially those in low-income countries, that there is a need for a better definition and employment of community engagement, as well as that community health workers, should be fully integrated into the health care system. Key enablers of PHC strengthening include equity-informed financing models to support PHC, health system and governance frameworks that differentiate multisectoral PHC from more discrete service-focused primary care, and governance mechanisms that strengthen linkages between policymakers, civil society, NGOs, community-based organizations, and private sector entities (Edelman *et al.*, 2021). Coordination and collaboration in these areas, however, remain dependent on a functioning health information system.

Country experiences with the pandemic in 2020 and beyond underscored the value of collecting reliable data. During PHCMI workshops conducted in 2020 and 2021, it was pointed out that starting the PHC performance assessment process had contributed to countries’ successes in tackling the pandemic and reinforced their abilities to respond to crises.

Although, WHO EMRO conducted two separate assessments during COVID on PHC: ‘The Role of PHC in COVID-19 response in 17 countries’ (WHO Regional Office for the Eastern Mediterranean, Role of PHC in COVID, 2020) and ‘The Role of the private health sector in COVID-19 response’ (WHO Regional Office for the Eastern Mediterranean, United Nations Children’s Fund, USAID, UNFPA, UNAIDS, UNHCR, Wonca East Mediterranean, n.d.), there is a need to continue showcasing the lessons learned from countries’ responses to the pandemic, highlight the importance of PHC and generate more political advocacy for strengthening PHC as a cornerstone of responsive and resilient health systems (WHO Regional Office for the Eastern Mediterranean, Role of PHC in COVID, 2020).

### Limitations

Major divergences dependent on the geopolitical and economic situations of countries created both challenges and opportunities resulting in a significant impact on the collection of data and the results. Countries with limited resources would often face challenges in finding available and reliable data at national and sub-national levels. The quality of data reported across the 124 indicators of the MIL presented difficulties. Some indicators were seen as irrelevant given a country’s context, and a flexible approach to the use of alternative indicators emerged as a major factor in the successful implementation of the initiative. Metadata for some of the indicators was not always clear and affected the understanding of the indicators. Some indicators deemed unclear or too complicated to calculate, including the antenatal quality score and the family planning quality score, were not reported by any country. While the use of commonly accepted international key indicators helped ensure standardization and comparability, comparisons between countries proved difficult given that indicators were not measured consistently across all countries, leading to suggestions that a core of indicators of common interest be identified. Understanding of the PHC approach and of the methodology of global health estimates as compared to the methodologies used at a national level to provide similar estimates was sometimes limited, contributing to the low uptake of global estimates and resulting in discrepancies between the values derived from national and global estimates. Rapid turnover of MOH officials contributed to delays in data collection. Other challenges included data fragmentation, poor data sharing mechanisms, and lack of up-to-date evidence for some measures. In many countries, data from the private sector was not reported, and confidentiality concerns made some countries unwilling to share all the necessary data.

Finally, many countries were still completing their assessments and moving towards the improvement phase when the emergence of the pandemic in early 2020 interrupted the process, reducing the availability of stakeholders as Ministries of Health were focused on combating the pandemic with shifting in the Ministries’ priorities. This resulted in delays in data collection, in endorsing profiles, and a reduction in in-person communication and access to information.

## Conclusion

*‘In order to improve the country’s service delivery system, it is necessary to measure the different dimensions of PHC based on standard and key indicators and plan for its improvement based on this assessment.’*

I.R. of Iran’s questionnaire response

The need to strengthen PHC in EMR cannot be overstated. Identified areas needing attention include the need for increasing the primary care workforce and appropriately distributing said workforce, additional training for primary care clinicians, improving the health information system, educating the population on the benefits of preventive care, and, possibly most important, the need for increased investment in the PHCsphere.

There is also a need to advocate for service delivery to be more tailored to the PHC approach and to encourage a more holistic view of the PHC approach as central to achieving UHC and health security (WHO Regional Office for the Eastern Mediterranean, Role of PHC in COVID, 2020). In short, there is a need to make PHC ‘everybody’s business’ and call on global, regional, and national leadership to voice their commitment (WHO Regional Office for the Eastern Mediterranean, PHCMI, n.d.).

The PHCMI initiative provided a regional foundation for assessing PHC performance and has opened a discourse for an improvement-based approach in all settings to advance UHC and health security. The resulting assessments have helped identify the strengths, weaknesses, and challenges faced by countries in the Region, explored a whole-of-government approach to advancing PHC, and provided countries a foundation on which to develop their improvement workplans.

The focus on the assessment of the health system’s ability to deliver quality primary care services rather than on the outcomes of primary care led to a broader appreciation of the PHC approach and furnished the basis for each country to develop a roadmap on how to make improvements as mentioned in the recent Regional Resolution as one of the seven main regional priorities for building health systems post-COVID (WHO Regional Office for the Eastern Mediterranean, EM/RC69/R.2, 2022).

## Supporting information

Kinder et al. supplementary material Kinder et al. supplementary material

## Supplementary material

The supplementary material for this article can be found at https://doi.org/10.1017/S1463423625000179
