# Supplementary material for: Take home messages from the implementation of the primary health care measurement and improvement (PHCMI) initiative in the WHO Eastern Mediterranean Region (EMR)
Source: Prim Health Care Res Dev. 2025 Mar 14;26:e30. doi: 10.1017/S1463423625000179 (PMC11955536; doi:10.1017/S1463423625000179)
Supplement: Kinder et al. supplementary material [file S1463423625000179sup001.pdf]

ANNEX 1: Master Indicator List

Number	Indicator	Quantitative/Qualitative	Notes/Definition	Numerator	Denominator	Usual Source	Alternate Source
1	A comprehensive national health sector policy, strategy or plan with goals and targets that includes all three components of a PHC approach exists and has been updated	Qualitative				National health policy, strategy or plan	N/A
2	Presence of UHC legislation inclusive of PHC	Qualitative				Legislative review	N/A
3	Participatory governance structures	Qualitative				Minutes of national policy, strategy or planning sessions	Legislative review (where participation legislated), national health policy, strategy or plan
4	Equity mainstreamed in health policy	Qualitative				National health policy, strategy or plan	
5	Existence of regulatory authorities for (HWF, facilities, EMP) for both public and private sectors	Qualitative				National health policy, strategy or plan	
6	Presence of quality improvement and assurance processes in the national health plan	Qualitative				National health policy, strategy or plan	
7	Adoption of HiAP approach and 2. Existing mechanism for multisectoral governmental coordination	Qualitative					
8	Inclusion of indicators on relevant social, economic, environmental and commercial determinants of health in NHPSP	Qualitative					
9	GDP per capita (PPP current international $)	Quantitative	Gross domestic product per capita converted to international dollars using purchasing power parity rates. An international dollar has the same purchasing power over GDP as the U.S. dollar has in the United States. Data are in current international dollars.	GDP is the sum of gross value added by all resident producers in the economy plus any product taxes and minus any subsidies not included in the value of the products. It is calculated without making deductions for depreciation of fabricated assets or for	Total population		
10	Population living in poverty (Under $1.90 int'l dollars / day)	Quantitative	Percentage of the population living in poverty, defined as living on less than $1.90 international dollars per day. An international dollar has the same purchasing power over GDP as the U.S. dollar has in the	Total population living on less than $1.90 international dollars per day	Total population		
11	Government health spending as percentage of GDP	Quantitative	Domestic General Government Health Expenditure as % of GDP measures current government expenditure on health, from domestic sources, relative to the country's GDP. Domestic General Government Health	Domestic General Government Health Expenditure	Gross Domestic Product (GDP)		
12	Total PHC spending per capita	Quantitative	Primary Health Care (PHC) expenditure monitors current health expenditure on a given set of health services defined within the System of Health Accounts 2011 (SHA 2011) framework. This includes government and non-government expenditures. The selected subset of health services includes general outpatient care, dental care, home-based curative care, outpatient and home-based long-term care, and preventive care (IEC, immunization, early disease detection, healthy condition monitoring, disease control programmed)[1]. To this subset of	PHC expenditure (in USD) - See SHA PHC Expenditure Tab	Total population	System of Health Accounts	Budgeting/expenditure data
13	% PHC expenditure from current health expenditure	Quantitative	Primary Health Care (PHC) expenditure monitors current health expenditure on a given set of health services defined within the System of Health Accounts 2011 (SHA 2011) framework. This includes government and non-government expenditures. The selected subset of health services includes general outpatient care, dental care, home-based curative care, outpatient and home-based long-term care, and preventive care (IEC, immunization, early disease detection, healthy condition monitoring, disease control programmed)[1]. To this subset of health services are added medical goods (medicines, glasses, hearing aids)1. Note that capital investments are excluded. Current health expenditure (CHE) refers to all health care goods and services used or consumed during a year by residents of a country. Note that capital investments are excluded.	PHC expenditure - See SHA PHC Expenditure Tab	Total health expenditure	System of Health Accounts	Budgeting/expenditure data
14	% PHC expenditure from domestic general Government health expenditure	Quantitative	Share of domestic general government health expenditure allocated to PHC	PHC expenditure - See SHA PHC Expenditure Tab	Domestic general government expenditure	System of Health Accounts	Budgeting/expenditure data
15	Domestic general government expenditure on PHC as a % of total PHC expenditure	Quantitative	[1] For more information, refer to the System of Health Accounts 2011	Domestic general government expenditure on PHC	Total PHC expenditure (See SHA PHC Expenditure Tab)	System of Health Accounts	Budgeting/expenditure data
16	Other sources of PHC expenditure (OOP, donor, etc.) as % of total PHC expenditure	Quantitative		Total (out-of-pocket or donor or other) expenditure on PHC	Total PHC expenditure (See SHA PHC Expenditure Tab)	System of Health Accounts	
17	% health workforce in primary care (by occupation)	Quantitative	[1] For more information, refer to the System of Health Accounts 2011	Number of PC HWF (doctors, nurses, midwives, community health workers etc. as per country specific assessment parameters) -	Total Number of HWF (doctors, nurses, midwives, community health workers, etc. as per country specific assessment parameters)	Health workforce accounts, personnel	
18	% primary care workforce specialized in family practice (by occupation)	Quantitative	Insert one row for each occupation analyzed, clarify if using data on licensing/registration or active practice	Number of primary care workforces specialized in family practice (doctors, nurses, midwives, community health workers, etc. as per country specific assessment parameters)	Total number of primary care HWF (doctors, nurses, midwives, community health workers, etc. as per country specific assessment parameters)		
19	Proportion of HWF in PHC have received minimum continuous professional education according to national requirements in the last year	Quantitative	Insert one row for each occupation analyzed, clarify if using data on licensing/registration or active practice	Number PC WF received minimum continuous professional education according to national requirements in the last year (doctors, nurses, midwives, community health workers etc. as per country specific	PC HWF (doctors, nurses, midwives, community health workers etc. as per country specific assessment parameters)		
20	Vacancy rate in PHC	Quantitative	Include notes on what occupations were analyzed	Number of vacant posts in primary care positions (in Full-Time Equivalents)	Total number of posts in primary care positions (in Full-Time Equivalents)		
21	Density of PHC by occupation (N/10,000 population)	Quantitative	Insert one row for each occupation analyzed,	Number of PC HWF by occupation (doctors,	Total population (standardized to per 10,000)		
22	% births registered	Quantitative		Number of births registered	All births		
23	% deaths registered	Quantitative		Number of deaths registered	All deaths		
24	Inclusion of section on PHC performance in annual health sector reporting	Qualitative				Annual health	
25	% public sector PHC that reports performance data	Quantitative	Consider reporting public and private separately as able (adding individual rows)	Total number of private and public sector facilities that report PC performance data	Total number of private and public sector PC facilities	Facility reports	
26	Presence and use of unique identifiers for patients	Qualitative or Quantitative		Number of primary care facilities that have and use unique identifiers	Total number of primary care facilities	Facility surveys	PHC policies, supervision reports
27	Presence of a comprehensive individual patient/family record	Qualitative or		Number of primary care facilities that have	Total number of primary care facilities	Facility surveys	PHC policies, supervision reports
28	Is there a functioning eHIS in the country?	Qualitative	Please clarify what types of functions are			eHealth	National digital health strategy
29	% PC facilities using eHIS.	Quantitative		Number of PC facilities using eHIS	Total number of PC facilities	Facility survey	eHealth observatory
30	% population that would have to travel more than 5 km/1 hour to arrive at	Quantitative		Number of patients that would have to travel	Total population	Facility registry,	Population survey
31	% PHC facilities with adequate WASH	Quantitative		Number of PC facilities with adequate WASH	Total number PC facilities	Facility survey	Facility registry, facility
32	% of PC facilities with rooms with auditory and visual privacy for patient consultations	Quantitative	Private room or screened off area available in main service area (usually the general outpatient service area), a sufficient distance from sites where providers/clients routinely may be, so that a normal conversation could be held without being overheard, and without the client being observed. (SARA)	Number of PC facilities with rooms with auditory and visual privacy for patient consultations	Total number PC facilities	Facility survey	Facility registry, facility maintenance records
33	% of PC facilities with communication equipment (phone or SW radio)	Quantitative	Functioning communication equipment. This will not include private cell phones unless the facility reimburses for cost of phone	Number of PC facilities with Communication equipment (phone or SW radio)	Total number PC facilities	Facility survey	Facility registry, facility maintenance records
34	% of PC facilities with access to computer with email/internet access	Quantitative	Facility has a functioning computer and has access to email/internet with internet working on the day of the survey (SARA)	Number of PC facilities with access to computer with email/internet access	Total number PC facilities surveyed	Facility survey	

35	% of PC facilities with standard precautions for infection prevention	Quantitative	Facilities with items for adherence to standard precautions in each/all assessed service area, facility level systems for safe final disposal of sharps and medical waste, system for sterilizing/high-level disinfecting equipment appropriate to the services offered, personal protective equipment, and	Number of PC facilities with standard precautions for infection prevention	Total number PC facilities		
36	% of PC facilities with PHC EML correlated to package of services delivered in PHC	Qualitative		Number of PHC facilities with PHC EML list correlated to package of services delivered in PHC	Total number PC facilities	PHC Strategy, National EML List, primary care formulary	
37	Proportion of facilities in which essential medicines are available (no stock		Relevant only to facilities that are intended	Number of primary care facilities that	Total number of PC facilities that dispense	Facility survey	Logistics management information
38	% PHC facilities with standard priority diagnostics and equipment available	Quantitative		Number of primary care facilities with standard priority diagnostics and equipment available according to national standards	Total number of primary care facilities	Facility Survey	Facility registry, facility maintenance records, supervision report
39	% of patients who get registered by PHC facilities	Quantitative		Number of patients registered at a PC facility	Total population	Facility survey	
40	% of PHC cases referred to secondary care	Quantitative		Number of PC cases referred to secondary	Total number of encounters in primary care	Facility survey	Referral registry, utilization records
41	Annual number of outpatient department visits per capita	Quantitative		Total number of OPD visits in last year	Total population	RHO	Facility survey, facility registry
42	% of PHC facilities that can provide mental health services	Quantitative	Specify what mental health services are available (medications, counselling, integrated/collocated behavioral therapy, for what conditions?)	Number of PHC facilities that can provide mental health services	Total number of primary care facilities	Mental health atlas	Facility survey, facility specifications
43	Number of consultations per health worker (physician, nurse, etc.) per day	Quantitative	Insert one row for each occupation analyzed	Number of consultations per health worker	Total number of work days in month	Facility survey	Facility registry, supervision reports
44	Gatekeeper Role for GPs / FPs	Qualitative	Is there a requirement (or incentive) for patients to first be seen by a primary care clinician prior to seeing a specialist?			National health policy, strategy or plan	
45	Formal Process exists for Referrals	Qualitative	Does a formal process exist to ensure			National health	
46	Evidence-based national guidelines/protocols/standards exist for the management of all priority causes of morbidity and mortality	Quantitative	Include information on priority causes of morbidity and mortality included in notes. Include information on which protocols are missing.	Number of evidence-based national guidelines/protocols/standards for the management of all priority causes of morbidity and mortality	Total number of priority causes of morbidity and mortality	National treatment protocol review	
47	Professionalized management at PC level	Quantitative					
48	Proportion of facilities with up-to-date performance reports in the last 6	Quantitative		Number facilities with up-to-date	Total number of primary care facilities		
49	% PC facilities with systems to support quality improvement	Quantitative		Number of PHC facilities with systems to	Total number of primary care facilities	Facility survey	Facility registry, supervision report
50	Diagnostic accuracy	Qualititative	Does a formal process exist to assess				
51	% PC facilities that monitor patient experience	Quantitative		Number of PHC facilities that monitor patient	Total number of primary care facilities	Facility survey	Facility registry, supervision report
52	Community/patient participation in facility management meetings						
53	Number of adverse events reported (immunization/medication)	Quantitative	Please clarify what types of events are included in adverse event reporting	Number of adverse events reported	Total number of adverse events	Available adverse reporting system	
54	% of PHC prescriptions that include antibiotics in out-patient clinics	Quantitative		Number PC prescriptions that include	Total number of PC prescriptions	Logistics	Pharmacy records, facility survey,
55	% of PHC prescriptions that include injectable medicines	Quantitative		Number PC prescriptions that include	Total number of PC prescriptions	Logistics	Pharmacy records, facility survey,
56	% of registered hypertension patients with BP <140/90 at last 2 follow up visits	Quantitative		Number patients with BP <140/90 at last 2 follow up visits	Total number of registered hypertension patients	Facility survey	Facility reports from chronic disease registry, supervision reports
57	% of registered diabetic patients with fasting blood sugar controlled at last 2	Quantitative		Number patients with fasting blood sugar	Total number of registered diabetic patients	Facility survey	Facility reports from chronic
58	% of registered NCD patients with 10 years cardiovascular risk recorded in	Quantitative		Number patients with 10 years	Total number of registered NCD patients	Facility survey	Facility reports from chronic
59	% of women who delivered and received at least once postnatal care within the first 40 days	Quantitative	If alternate time frame used, please specify	Number of women who delivered in past 6 months who received at least once postnatal care within the first 40 days	Total number of women who delivered in last 6 months	Facility registry	Facility reports from chronic disease registry, supervision reports
60	% of substance users including tobacco users in receipt of brief intervention	Quantitative	Specify time frame analyzed	Number of substance users including tobacco	Total number of substance users seen	Facility registry	Facility reports from chronic
61	% of under 5 children that had weight and height measured in past 1 year	Quantitative		Number of under 5 children that had weight	Total number of children under 5 seen		
62	Hospital admissions for ambulatory sensitive conditions diagnoses	Quantitative	Age-standardized acute care hospitalization rate for conditions where appropriate ambulatory care may prevent or reduce the need for admission to hospital, per 100,000 population. Conditions include hypertension, congestive heart failure, diabetes mellitus, asthma and chronic obstructive pulmonary disease	Number of acute care hospitalizations for ambulatory care sensitive conditions (ACSCs).<br>Inclusions<br>• Admission to an acute care hospital for an individual in the denominator<br>• Admission to an acute care hospital with one of the<br>following as most responsible diagnosis:<br>– Chronic obstructive pulmonary disease<br>– Asthma<br>– Heart failure and pulmonary edema<br>– Hypertension<br>– Diabetes<br>Exclusions<br>• Individual died before discharge<br>• Admission category recorded as newborn or stillbirth	Mid-year population age 75 and younger, per 100,000 (age adjusted).<br>Inclusions<br>• Age of individual is younger than 75 years	Discharge records	Administrative data
63	Children under 5 who are stunted, wasted, overweight, obese	Quantitative		Number of children under 5 who are stunted,	All children under 5		
64	Exclusive Breastfeeding 0-5 months (%)	Quantitative	Proportion of infants 0–5 months of age who	Infants 0–5 months of age who received only	Infants 0–5 months of age)	Population	
65	Cervical cancer screening rates	Quantitative	Proportion of women between the ages of	Number of women aged 30-49 years who	All female respondents aged 30-49 years.	Population	Facility registry, facility survey
66	Vaccination of Measles2 and DPT3	Quantitative		The number of individuals in the target group for each vaccine that has received the last recommended dose in the basic series	The total number of individuals in the target National population-based survey, routine facility information system group for each vaccine.	National population-based survey, routine facility information system	
67	% households with adequate WASH [6.1.1/6.2.1]	Quantitative		Number of households with adequate WASH	All households		
68	% households cooking with clean fuel [7.1.2]	Quantitative		Number of households cooking with clean	All households		
69	% children under 5 years of age who are developmentally on track [4.2.1]	Quantitative	The proportion of children under 5 years of	The number of children under the age of five	Total number of children under the age of	Population	
70	Physical inactivity in adults	Quantitative	Percentage of adults aged 18+ years not meeting any of the following criteria:<br>– 150 minutes of moderate-intensity physical activity per week<br>– 75 minutes of vigorous-intensity physical activity per week<br>– an equivalent combination of moderate-	Number of respondents where all 3 of the following criteria are true:<br>(1) Weekly minutes* of vigorous activity < 75 mins.<br>(2) Weekly minutes* of moderate activity < 150 mins.<br>(3) Weekly MET-minutes** < 600.	All respondents aged 18+ years in the survey.	Population-based (preferably nationally representative) survey	FAO or UN Statistical Data Set
71	Proportion of population subjected to physical, psychological or sexual	Quantitative		Number of individuals subjected to physical,	Total population deaths		
72	Adult mortality rate 15-60 years	Quantitative	the probability that those who have reached age 15 will die before reaching age 60 (shown per 1,000 persons)	This is based on a “synthetic cohort”: current life-table mortality rates are applied to the current cohort of 15 year olds, assuming no changes in mortality.		World Development Indicators	
73	Adolescent mortality rate	Quantitative	Number of deaths among adolescents (10-19	Mortality rates by age and sex for WHO		Global Health	
74	U5 Mortality Rate	Quantitative	Probability of dying between birth and	Data correspond to mid-year estimated		Global Health	
75	Infant Mortality Rate	Quantitative	Infant mortality rate is the probability of a	The Inter-agency Group for Child Mortality		Global Health	
76	Neonatal mortality rate	Quantitative	Number of deaths during the first 28	Number of deaths among life births during	Live births in a given year (expressed per	Civil Registration	Population survey, Household
77	Total fertility rate	Quantitative	The average number of children a	The average number of children a		Global Health	Civil Registration, Population
78	Met need for family planning [3.7.1]	Quantitative	Proportion of women of reproductive age	Percentage of women of reproductive age	The denominator is the total demand for	Multi-country	
79	Maternal mortality ratio [3.1.1]	Quantitative	The maternal mortality ratio (MMR) is the	Maternal deaths	Number of live births (expressed per	Vital registration,	Sample registration systems;
80	Life Expectancy at Birth (years)		The average number of years that a newborn could expect to live if he or she were to pass through life exposed to the sex- and age-specific death rates prevailing at			Vital registration, health service records, household	
81	Premature NCD mortality [3.4.1]	Quantitative	Probability of dying between the exact ages	Age-specific death rates for the combined		WHO NCD	
82	Causes of Death	Quantitative	Causes of death disaggregated by	Total number of deaths by cause in a given	Total number of deaths in a given year	Vital registration,	
83	Suicide rate [3.4.2]	Quantitative	Number of suicide deaths divided by the	Number of suicides	Total population deaths	Vital registration	Sample registration systems;
84	Coverage of RMNCH by mothers' education	Quantitative	Difference in RMNCH coverage index for households with mothers that have completed secondary level education versus those without secondary level education.			WHO Health Equity Monitor/Population Survey	
85	Average availability of 5 Tracer RMNCH Services		Average of the percentage of five maternal and child health services (child, vaccination, family planning, antenatal care, and prevention of mother-to-child transmission of HIV) provided at each facility surveyed.			SPA	
86	U5 mortality by residence	Quantitative	Difference in under 5 mortality rates between residents of urban areas and rural areas. Probability (expressed as a rate per 1000 live births) of a child born in a specific year or period dying before reaching the age			WHO Health Equity Monitor/Population Survey	

87	% population with impoverishing health expenditure [3.8.2]	Quantitative					
88	IHR Core Capacity Index / JEE	Quantitative/Q				IHR Score	JEE Score
89	Disaster related death rate [1.5.1]	Quantitative	This indicator measures the number of	This indicator, X, is calculated as a simple	Total population	Sendai	
90	Obesity prevalence	Quantitative	Percentage of defined population with a	number of persons aged 18+ with BMI of 30	Total population aged 18+ surveyed	Population	Other population based estimate
91	DM prevalence	Quantitative	Age-standardized prevalence of raised blood	persons aged 18+ years (defined as fasting	Total population aged 18+ surveyed	Population	Other population based estimate
92	HTN prevalence	Quantitative	Percent of population aged 18+ with raised blood pressure (systolic blood pressure ≥ 140 OR diastolic blood pressure ≥ 90 OR on medication for raised blood pressure).	Number of population aged 18+ with raised blood pressure (systolic blood pressure ≥ 140 OR diastolic blood pressure ≥ 90 OR on medication for raised blood pressure).	Total population aged 18+ surveyed	Population Survey (STEPS)	Other population based estimate (UN)
93	Tobacco use [3.A.1]	Quantitative	The indicator is defined as the percentage of	Number of current tobacco users aged 15+	All respondents of the survey aged 15+ years.	Population	Other population based estimate
94	Household and ambient air pollution [3.9.1]						
95	Road traffic injuries [3.6.1]			Number of road traffic injuries	Total population		
96	Homicide [16.1.1]			Number of homicides	Total population d		
97	% population who believe decision making is inclusive [SDG 16.7.2]			Number of individuals in the population who	Total populations		
98	Perceived access Barriers due to treatment costs	Quantitative	Access barriers due to treatment cost	Number of women who report specific	Number of women interviewed	Population	
99	Perceived access Barriers due to distance	Quantitative	Access barriers due to distance measures the percent of women who self-report that the distance they have to travel to receive medical advice or treatment is a big problem.	Number of women who report the distance to the health facility as a big problem in getting medical advice or treatment when sick	Number of women interviewed	Population Survey (DHS)	
100	DPT3 Dropout rate	Quantitative	Diphtheria-tetanus-pertussis (DTP) dropout rate is the percent of children who do not receive the full three doses of DTP vaccination after receiving the initial dose.	[DTP1 Immunization Coverage - DTP3 Immunization Coverage]	DTP1 Immunization Coverage	WHO/UNICEF (based on country reported administrative data and household	
101	Proportion of Family planning, ANC, and sick child visits over 10 minutes	Quantitative	The proportion of antenatal care, family	The number of observed client visits for	The total number of ANC, FP and sick child	Facility Survey	Supervision reports
102	Provider absence rate	Quantitative	Provider absence rate measures the number of clinical staff actually present at a facility compared to the expected number of staff at a given time.	Number of health professionals that are not off duty who are absent from the facility on an unannounced visit	Ten randomly sampled workers who are supposed to be on duty at the facility on the day of the assessment. The only health workers that are removed from the denominator are those on shift work (i.e., not present because it is not their shift) or those on long absences due to long term sick	Facility Survey	Supervision reports
103	Antenatal care coverage (4+ visits)	Quantitative	Antenatal care coverage (4+) visits is the	The number of women aged 15-49 surveyed	Total number of women aged 15-49 with a	UHC Index	
104	Care seeking for suspected child pneumonia	Quantitative	Percentage of children under 5 years of age	Number of children (0-59 months) with	Number of children (0-59 months) with	DHS/MICS	
105	Children under 5 with diarrhea receiving ~~ORS~~	Quantitative	The percent of children with diarrhea, a	Number of children under 5 years of age with	Total number of children aged 0–59 months	DHS/MICS	
106	Average availability of diagnosis and management of 3 tracer NCDs (diabetes, CRD, CVD)	Quantitative	Proportion of non-combinable disease services provided (diabetes, chronic respiratory disease, and chronic cardiovascular disease) across all facilities.	Number of facilities offering 3 NCD services	All PHC facilities surveyed	SPA	
107	Average availability of services for 3 Tracer Communicable Diseases (STI, TB, HIV)	Quantitative	Average of the percentage of service for three tracer communicable diseases (HIV, STI, and TB) provided at each facility surveyed.	Number of facilities offering 3 primary ID services	All PHC facilities surveyed	SPA	
108	Coverage of DPT3 Immunizations	Quantitative	Diphtheria-tetanus-pertussis (DTP) coverage measures the percent of one-year-olds who have received three doses of the combined diphtheria, tetanus toxoid and pertussis vaccine in a given year.	Number of children of aged 12 months surveyed who have received three doses of the combined diphtheria, tetanus toxoid and pertussis vaccine in a given year	Total population of children aged 12 months surveyed		
109	TB treatment success	Quantitative	Percentage of tuberculosis (TB) cases successfully treated (cured plus treatment completed) among TB cases notified to national health authorities during a specified period, usually one year.	Number of TB cases registered in a specified time period that were successfully treated with or without bacteriological evidence of success	Total number of TB cases registered in the same period	WHO Tuberculosis Program (based on country reported administrative data and surveys)	
110	Proportion of caregivers who were told the sick child diagnosis	Quantitative	Proportion of observed sick child visits	Number of sick child visits observed where	Total number of sick child visits observed	SPA	
111	Antenatal care quality score based on WHO guidelines		Average quality score for observed antenatal care visits based on WHO antenatal care guidelines.			SPA	
112	Family planning quality score based on WHO guidelines		Average quality score for observed family planning visits based on WHO Family Planning guidelines.			SPA	
113	Demand for family planning satisfied with modern methods	Quantitative	Proportion of married or in-union women of reproductive age (aged 15-49 years) who are	Number of married or in-union women of reproductive age (15–49 years old) who are	Total demand for family planning (the sum of contraceptive prevalence (any method)	UHC Index	
114	Sick child quality score based on IMCI guidelines		Average quality score for observed sick child visits based on the WHO's Integrated Management of Childhood Illness (IMCI) program guidelines.			SPA	
115	Adherence to clinical guidelines	Quantitative	Adherence to clinical guidelines measures the number of relevant history and examination questions asked by a provider during a clinical encounter compared to the	Total number of relevant history and examination questions asked by the provider	Total number of relevant history and examination questions that should have been asked by the provider	SDI	
116	Diagnostic accuracy	Quantitative	Diagnostic accuracy measures the number of cases that are correctly diagnosed out of the number of patients examined, as observed through clinical vignettes on multiple common conditions, including pulmonary tuberculosis, pneumonia, acute diarrhea, diabetes, and malaria with anemia.	For each clinical case, a score of one is assigned for each clinical case if the diagnosis is mentioned. The numerator is the sum of the total number of correct diagnoses identified. Where multiple diagnoses were provided by the clinician, the diagnosis is coded as correct as long as it is mentioned, irrespective of what other alternative diagnoses were given	Total number of clinical cases tested	SDI	
117	Adequate waste disposal	Quantitative	Average score (out of 3) on adherence to	Total of the average number of the 3 waste	Total number of facilities	SPA	
118	Proportion of rooms with all infection control items - MAYBE REPEAT OF #32	Quantitative	Proportion of rooms (family planning, sick	Number of rooms where all the infection	Total number of rooms observed	SPA	
119	People living with HIV receiving anti-retroviral treatment	Quantitative	Percentage of people living with HIV	Number of adults and children who are	Estimated number of adults and children	Global Health	
120	Use of insecticide-treated nets (ITN) for malaria prevention	Quantitative	Percentage of population in malaria-	Number of people in malaria-endemic areas	Total number of people in malaria endemic	UHC Index	
121	Prevalence of raised blood pressure (age-standardized estimate)	Quantitative	Age-standardized prevalence of raised blood pressure among persons aged 18+ years (defined as systolic blood pressure ≥ 140 mmHg and/or diastolic blood pressure ≥ 90 mmHg).	Number of respondents with systolic blood pressure ≥ 140mmHg or diastolic blood pressure ≥ 90mmHg	All survey respondents with a valid measurement	UHC Index	
122	Tuberculosis cases detected and treated (%)	Quantitative	Number of new and relapse cases of tuberculosis (TB) that were notified and treated successfully in a given year, divided by the estimated number of incident TB cases in the same year, expressed as a percentage.			Joint World Bank/WHO "Tracking Universal Health Coverage: 2017 Global Monitoring Report"<br><br>WHO's 100 Core Indicators - Page	

	INDICATORS, METADATA and VALUES
**Notes:**	This sheet represents the list of indicators that can be used for the PHC
**Additional**	**RED** indicates a PHC Core Indicator (N = 14)
	Green fill indicates a Vital Signs Profile Indicator
	If data is not available for a given indicator please indicate this in column J
	If an alternate indicator is suggested, please include suggested indicator and
	Orange fill indicates an SDG indicator. The numbers included in brackets
**Indicator**	Facility Survey = SARA, Harmonized Facility Survey, Other
	Population Survey = DHS, MICS, STEPS, Other
